# PREVALENCE OF OSTEOPOROSIS AND HYPOVITAMINOSIS D AT SIRIRAJ METABOLIC BONE DISEASE CLINIC

**DOI:** 10.1590/1413-785220172506174133

**Published:** 2017

**Authors:** AASIS UNNANUNTANA, POJCHONG CHOTIYARNWONG

**Affiliations:** 1. Department of Orthopedic Surgery, Faculty of Medicine Siriraj Hospital, Mahidol University, Bangkok, Thailand.

**Keywords:** Osteoporotic fracture, Bone diseases, metabolic, Bone density, Vitamin D, Osteoporosis., Fraturas por osteoporose, Doenças ósseas metabólicas, Densidade óssea, Vitamina D, Osteoporose*.*

## Abstract

**Objective:**

To identify the prevalence of osteoporosis and hypovitaminosis D among patients at the Siriraj Metabolic Bone Disease (MBD) Clinic, and to compare initial vitamin D levels in patients with and without a history of fragility fractures.

**Methods:**

Medical records of patients who attended our MBD clinic between 2012 and 2015 were retrospectively reviewed. Patient baseline demographic, clinical, bone mineral density (BMD), and laboratory data were collected and analyzed. Osteoporosis was diagnosed when patients had a BMD T-score <-2.5 or presented with fragility fractures.

**Results:**

There were 761 patients included in this study. Of these, 627 patients (82.4%) were diagnosed with osteoporosis and 508 patients (66.8%) had fragility fractures. Baseline serum 25-hydroxyvitamin D (25(OH)D) levels were available in 685 patients. Of these, 391 patients (57.1%) were diagnosed with hypovitaminosis D. When evaluated only in patients with fragility fractures, the average initial 25(OH)D level was 28.2±11.6 ng/mL, and the prevalence of hypovitaminosis D was 57.6%.

**Conclusion:**

A high prevalence of osteoporosis and hypovitaminosis D was found among patients at our clinic; two-thirds of patients had a history of fragility fractures, and no difference in initial 25(OH)D levels was seen between patients with and without fragility fractures. **Level of** **Evidence III, Retrospective Study** ***.***

## RESUMO

**Objetivo:**

Identificar a prevalência de osteoporose e hipovitaminose D entre os pacientes na Siriraj Metabolic Bone Disease (MBD) Clinic e comparar o nível inicial de vitamina D em pacientes com e sem história de fratura por fragilidade óssea*.*

**Métodos:**

Os prontuários de pacientes atendidos em nossa clínica MBD durante o período de 2012 a 2015 foram analisados retrospectivamente*.* Os dados demográficos, clínicos, densidade mineral óssea (DMO) e os dados laboratoriais basais foram coletados e analisados*.* A osteoporose foi diagnosticada quando os pacientes tinham DMO com escore T≤ *-*2,5 ou fraturas por fragilidade óssea*.*

**Resultados:**

Foram incluídos 761 pacientes dos quais, 627 pacientes (82,4*%*) foram diagnosticados com osteoporose e 508 (66,8*%*) tinham fraturas por fragilidade*.* O nível sérico basal de 25*-*hidroxivitamina D (25(OH)D) estava disponível para 685 pacientes*.* Desses, 391 pacientes (57,1*%*) foram diagnosticados com hipovitaminose D*.* Quando avaliado apenas em pacientes com fratura por fragilidade óssea, o nível inicial médio de 25*(*OH*)*D foi de 28,2 ± 11,6 ng*/*ml e a prevalência de hipovitaminose D foi de 57,6*%.*

**Conclusão:**

Encontrou-se alta prevalência de osteoporose e hipovitaminose D entre os pacientes de nossa clínica, sendo que dois terços deles tinham história de fratura por fragilidade óssea e nenhuma diferença no nível basal de 25*(*OH*)*D entre pacientes com e sem fratura por fragilidade. **Nível de Evidência III, Estudo Retrospectivo**

## INTRODUCTION

As people age, their chance of sustaining a fragility fracture increases. Approximately 50% of women and 20% of men will have a fragility fracture once in their lifetime.^1^ ^,^ ^2^ Wade et al.^3^ estimated the combined incidence rate of non-traumatic fracture in Japan, Australia, and ten countries in North America and Europe to be approximately 5.2 million, most of these in women. The treatment-related cost of fragility fracture is high. In Europe, the total direct costs of treating osteoporotic fracture was reported to be 32 billion euros per year,^4^ and the total cost of treating osteoporotic fractures in the United States in 2002 was 20 billion USD.^5^ However, previous studies have reported a surprisingly low rate of osteoporosis treatment in elderly individuals with fragility fractures, approximately 20%.^6^

The Department of Orthopedic Surgery at the Siriraj Hospital Faculty of Medicine was established in 1964, and after years of development and planning, the Siriraj Metabolic Bone Disease (MBD) Clinic was formally established in March 2012. The objectives of this special clinic are to provide diagnosis, treatment, and follow-up care to patients with metabolic bone diseases (particularly osteoporosis and osteomalacia); to teach medical residents and fellows the principles of metabolic bone disease and the application of treatment protocols; to conduct research in metabolic bone diseases; and to follow elderly patients with low-energy hip fractures as a part of the fracture liaison service at Siriraj Hospital. Since 2012, over 900 patients have sought treatment at the Siriraj MBD clinic.

The aims of this retrospective study were to evaluate the prevalence of osteoporosis and hypovitaminosis D in patients who attended the Siriraj MBD clinic during 2012 to 2015, and to compare the initial laboratory values in patients with and without a history of fragility fracture.

## MATERIALS AND METHODS

After receiving institutional review board approval (approval number Si252/2016), the authors retrospectively reviewed medical records from patients who sought treatment at the Siriraj MBD clinic from March 2012 to December 2015. Because of the retrospective methodology, consent forms were not deemed necessary by the institutional review board. Criteria for accepting patients to the Siriraj MBD clinic include clinical risk factors for osteoporosis, history of fragility fracture, and/or diagnosis of other types of metabolic bone diseases such as osteomalacia and Paget’s disease. Patients meeting one or more of these criteria were referred to our clinic and included in this retrospective study. Patients with incomplete data and/or pathologic fractures were excluded. Once they were enrolled in the MBD clinic, all information related to long-term management of the patient’s disease was obtained and recorded in the Siriraj MBD clinic registry. 

Patient information in the clinic registry is categorized into three sections: the first includes general patient information including risk factors for osteoporosis, the second includes history of falls, underlying diseases, and current medication, and the third includes laboratory testing and treatments given or prescribed at each follow-up visit. The subset of patients with a history of fragility fracture was also evaluated in a subgroup analysis. Fragility fracture is defined as any fracture that occurs spontaneously after a physiological load, such as fractures after falls from a standing height or less.^7^

### Bone mineral density (BMD) 

BMD was measured by Dual Energy X-ray Absorptiometry (DEXA) in the posteroanterior lumbar spine and proximal femur using the standard protocol provided by the manufacturer (Lunar Prodigy; GE Healthcare, Little Chalfont, United Kingdom). The average T-score for the lumbar spine from L1 to L4 levels was calculated. If there was any evidence of compression fracture or degenerative changes in this area, an alternative BMD T-score was calculated from at least two consecutive levels between L1 to L4 and used. If at least two consecutive levels of the lumbar spine were not available, that case was then classified as having an uninterpretable BMD at the lumbar spine, and BMDs of the femoral neck and total hip were used instead. Indications to screen for BMD were based on the Thai Osteoporosis Foundation guidelines.^8^ According to the WHO definition, osteoporosis is diagnosed when a patient’s BMD T-score is equal to or lower than -2.5.^9^ However, patients who had fragility fractures were diagnosed with osteoporosis regardless of their BMD level.

### Laboratory investigations

Fasting blood samples were obtained and sent for analysis at our hospital’s central laboratory. Serum 25-hydroxyvitamin D (25(OH)D and parathyroid hormone levels were measured using the chemiluminescence technique. Normal serum vitamin D level was defined as serum 25(OH)D level >30 ng/mL. Low serum vitamin D level (hypovitaminosis D) was subcategorized as either vitamin D insufficiency (20-29 ng/mL) or vitamin D deficiency (<20 ng/mL).^10^

Other baseline laboratory investigations included blood urea nitrogen (BUN), creatinine, total calcium, phosphorus, and albumin. Estimated glomerular filtration rate (eGFR) was calculated using the Chronic Kidney Disease Epidemiology Collaboration (CKD-EPI) equation.^11^ Baseline laboratory tests were compared between patients with and without a history of fragility fracture.

### Statistical analysis

All statistical analyses were performed using SPSS Statistics version 18.0 (SPSS, Inc., Chicago, IL, USA). Data are shown as number and percentage for categorical variables and mean ± standard deviation (SD) for continuous variables. Differences in baseline demographic data and clinical characteristics between patients with and without a history of fragility fracture were evaluated using Student’s t-test for continuous data and the chi-squared test for categorical data. A p-value <0.05 was regarded as statistically significant.

## RESULTS

From March 2012 to December 2015, 761 patients sought treatment and became patients at the Siriraj MBD clinic. Six hundred and twenty-seven patients (82.4%) were diagnosed with osteoporosis (based on T-score <-2.5 or positive history of fragility fracture). Of these 627 patients, 508 patients (81.0%) had fragility fractures. There were 708 patients with baseline BMD results available; of these, 59.6% were diagnosed with osteoporosis based on a BMD T-score <-2.5, 34.5% were diagnosed with osteopenia (T-score between -1.0 and -2.5), and 5.9% of patients had normal BMD (T-score >-1.0). In a subgroup analysis of patients with a history of fragility fracture and baseline BMD results (480 patients), we found that 303 patients (63.1%) had a BMD T-score <-2.5.

Patient demographic data and clinical risk factors for osteoporosis are shown in Table 1. The mean age of all patients was 72.0 years, and 90.8% were female. The average patient BMI was 23.0 kg/m^2^ (22.4 kg/m^2^ in men and 23.1 kg/m^2^ in women). In the subgroup of patients with a history of fragility fracture, mean patient age was 74.7 years. The mean BMI in fragility fracture patients was 23.1 kg/m^2^, and 88.8% were female. As for risk factors for osteoporosis, 75% of patients received calcium supplementation and 45.4% received vitamin D supplementation prior to their first visit to the Siriraj MBD clinic. Fewer than 10% of patients had a history of smoking, alcohol consumption, or family history of osteoporosis.

**Table 1:** Demographic data and clinical risk factors for osteoporosis in all patients and in patients with fragility fractures at the Siriraj MBD clinic.

Data and risk factors	All patients (N=761)	Fragility fracture patients (n=508)
Sex (female)	691 (90.8%)	451 (88.8%)
Age (years)	72.0±11.0	74.7±9.6
BMI (kg/m^2^)	23.0±4.1	23.1±4.4
**History of**		
Steroid use	97 (12.8%)	59 (11.7%)
Calcium supplementation	569 (75.0%)	377 (74.5%)
Vitamin D supplementation	343 (45.4%)	244 (48.3%)
Proton pump inhibitor use	248 (32.7%)	175 (34.6%)
Smoking	19 (2.5%)	16 (3.2%)
Alcohol consumption	21 (2.8%)	15 (3.0%)
Bisphosphonate use	208 (27.4%)	113 (22.3%)
Familial osteoporosis	65 (8.6%)	33 (6.5%)

Data presented as mean±standard deviation (SD) for continuous variables or frequency (percentage) for categorical variables.

Baseline laboratory investigations are shown in Table 2. Six hundred and eighty-five patients had serum 25(OH)D levels available for analysis. Among these patients, the mean serum 25(OH)D level was 28.4 ng/mL, which categorized the overall group as having vitamin D insufficiency. When we compared baseline serum 25(OH)D level between patients with and without a history of fragility fracture, we observed no statistically significant difference between groups (p=0.469). However, BUN and creatinine levels were higher, and serum calcium, albumin, phosphorus, and estimated glomerular filtration rate were lower in the fragility fracture group than in the group without fragility fractures. We also observed a trend toward lower parathyroid hormone levels in the fragility fracture group (p=0.084). When we compared serum 25(OH)D levels between patients with and without fragility fracture, there was no significant difference in the proportion of patients with low serum 25(OH)D levels (p=0.201). (Table 3)

**Table 2:** Initial laboratory testing for of all patients and patients with and without fragility fractures at the Siriraj MBD clinic.

Laboratory test	Laboratory reference range	All patients (N=761)	Fragility fracture patients (n=508)	Patients without fragility fractures (n=253)	p-value
Total calcium (mg/dL)	8.6-10.0	9.2±0.5	9.2±0.5	9.3±0.5	0.007*
Corrected total calcium (mg/dL)	8.6-10.0	9.1±0.7	9.0±0.8	9.2±0.9	0.001*
Albumin (g/dL)	3.5-5.5	4.0±0.5	3.9±0.5	4.2±04	<0.001*
Phosphorus (mg/dL)	2.5-4.5	3.5±1.7	3.4±0.5	3.6±0.5	0.001*
Parathyroid hormone (pg/mL)	15-65	51.5±23.2	50.5±23.6	53.7±22.1	0.084
25(OH)D (ng/mL)	≥30	28.4±11.3	28.2±11.6	28.8±10.7	0.469
BUN (mg/dL)	6-20	14.8±6.8	15.3±7.4	13.9±5.3	0.004*
Creatinine (mg/dL)	Female (0.51-0.95)	0.88±0.7	0.91±0.8	0.81±0.3	0.020*
	Male (0.67-1.17)	1.27±0.8	1.35±0.8	0.93±0.3	0.072
eGFR (mL/min/1.73m^2^)		53.4±23.2	49.9±22.4	60.4±23.3.	<0.001*

Data presented as mean±standard deviation (SD). *p-value less than or equal to 0.05 indicates statistical significance.

**Table 3:** Baseline 25(OH)D levels of patients with and without fragility fractures.

Baseline 25(OH)D level	Fragility fracture patients (n=463)	Patients without fragility fractures (n=222)	p-value
Deficiency (<20 ng/mL)	110 (23.8%)	40 (18%)	0.201
Insufficiency (20-29 ng/mL)	156 (33.7%)	85 (38.4%)	
Sufficiency (≥30 ng/mL)	197 (42.5%)	97 (43.7%)	

Data presented as number (percentage). *p-value less than or equal to 0.05 indicates statistical significance.

## DISCUSSION

Metabolic bone diseases (MBDs) are a group of bone disorders caused by abnormalities in calcium, phosphorus, magnesium, and vitamin D metabolism.^12^ These disorders need to be differentiated from genetic bone disorders, since many MBDs are treatable. Over the last twenty years, a vast amount of valuable information has been discovered regarding cellular and molecular biology, pharmacology, and genetics. Accordingly, the pathophysiology of many MBDs is now better understood, with significant improvements in patient care as a result.

Domrongkitchaiporn^13^ reviewed the prevalence of MBDs in Thailand in 2005 and reported the four most common MBDs to be renal tubular acidosis type 1, systemic fluorosis, thalassemia, and osteoporosis. Taechakraichana et al.^14^ and Limpaphayom et al.^15^ reported that 10% and 20% of women aged over 40 years were diagnosed with osteoporosis using hip BMD data and lumbar spine BMD data, respectively. In this study, we found a prevalence of osteoporosis at the Siriraj MBD clinic approximately 80%. The mean age of subjects in our study was 72.0 years, which is much higher than the mean age reported in the study from Limpaphayom et al.^15^ In addition, we found that two-thirds of patients at our MBD clinic had a history of fragility fracture. This finding reflects the nature of our patient population, with most of our patients referred from orthopedic surgeons at our center. The most common MBD we encountered at the Siriraj MBD clinic during the study period was osteoporosis. 

A comparison of baseline laboratory investigations between patients with and without a history of fragility fracture revealed statistically significant differences for many laboratory tests. (Table 2) However, the mean scores for each of those significantly different tests were still within the normal ranges for each test. This information suggests that, while statistically significant, these differences may not always be clinically relevant. 

Several strategies have been developed to increase the rate of osteoporosis treatment (especially after osteoporotic fracture), including the American Orthopedic Association’s Own the Bone^®^ initiative^16^ and Capture the Fracture^®^ - a best practice framework tool.^17^ The objectives of these strategies are to raise awareness among physicians, establish a proper treatment care plan, and promote long-term follow-up for osteoporosis patients, especially after fracture fixation. The Own the Bone^®^ initiative was launched as a pilot project in 2005. This quality improvement tool was developed to stimulate behavioral changes in both physicians and patients after low-energy fractures.^16^ The Capture the Fracture^®^ campaign was launched in 2012 by the International Osteoporosis Foundation (IOF) to substantially reduce the incidence of secondary fractures worldwide. Both of these programs were created to improve rates of long-term patient follow-up and increase the rate of medical treatment to prevent future fractures. The Siriraj MBD clinic was established, in part, to follow patients with fragility fractures, and to function as part of the fracture liaison service in the Capture the Fracture^®^ campaign.

As a component of non-pharmacologic treatment, calcium and vitamin D supplementation should be administered to all patients in this population. Our study found that the majority of patients treated at the Siriraj MBD clinic had osteoporosis and low vitamin D levels. Of 508 patients with fragility fractures, 57.6% had hypovitaminosis D. This finding suggests that physicians should increase their awareness regarding the severe health implications associated with fragility fractures, and that a more effective prevention policy is necessary. A MBD clinic can also be used as a tool to follow patients after fragility fractures as part of the fracture liaison service. 

This study has several limitations that can be mentioned. First, like all retrospective studies this study was subject to inherent biases in patient selection. Second, we did not have and were not able to include accurate information regarding patient dietary intake of vitamin D. It is therefore possible that some patients may have received vitamin D supplementation that was higher than planned or estimated. Lastly, this is a single-center study that was conducted at Thailand’s largest tertiary care center, which is located in Bangkok. As such, our findings may not be applicable to different centers or other regions of the country. 

## CONCLUSION

A high prevalence of osteoporosis and hypovitaminosis D was found among patients who attended the Siriraj MBD clinic, with almost two-thirds of patients having a history of fragility fracture. No difference was observed for initial 25(OH)D level between patients with and without fragility fractures. 
